# Intrinsic Sensing and Evolving Internal Model Control of Compact Elastic Module for a Lower Extremity Exoskeleton

**DOI:** 10.3390/s18030909

**Published:** 2018-03-19

**Authors:** Likun Wang, Zhijiang Du, Wei Dong, Yi Shen, Guangyu Zhao

**Affiliations:** 1 State Key laboratory of Robotics and System, Harbin Institute of Technology , Harbin 150080, China; likunwang@hit.edu.cn (L.W.); duzj01@hit.edu.cn (Z.D.); 2 School of Astronautics, Harbin Institute of Technology, Harbin 150080, China; shen@hit.edu.cn; 3 Weapon Equipment Research Institute, China Ordnance Industries Group, Beijing 102202, China; guangyuzhao1980@gmail.com

**Keywords:** series elastic module, human–robot interaction, evolving internal model control, distributed Gaussian process, force control, exoskeleton

## Abstract

To achieve strength augmentation, endurance enhancement, and human assistance in a functional autonomous exoskeleton, control precision, back drivability, low output impedance, and mechanical compactness are desired. In our previous work, two elastic modules were designed for human–robot interaction sensing and compliant control, respectively. According to the intrinsic sensing properties of the elastic module, in this paper, only one compact elastic module is applied to realize both purposes. Thus, the corresponding control strategy is required and evolving internal model control is proposed to address this issue. Moreover, the input signal to the controller is derived from the deflection of the compact elastic module. The human–robot interaction is considered as the disturbance which is approximated by the output error between the exoskeleton control plant and evolving forward learning model. Finally, to verify our proposed control scheme, several experiments are conducted with our robotic exoskeleton system. The experiment shows a satisfying result and promising application feasibility.

## 1. Introduction

In the past two decades, several types of prostheses, orthoses, and exoskeletons have been developed to meet the demand for human-limb assistance [1,2,3], interface of virtual reality [4,5], and strength augmentation [6] owing to dramatic progress in computing, sensing, as well as control. Thus, various studies have been devoted to developing novel compact mechanical structures and corresponding control methodologies.

To follow the human intention efficiently (i.e., discrete or rhythmic movements), the sensing system should provide full information for the exoskeleton controller. A feasible design of the Hybrid Assistive Limb exoskeleton [7] focuses on the electromyographical (EMG) signals. Besides, the human intention is collected by applying a pattern recognition technique. However, the main limitation is that the EMG signals suffer heavily from unwanted noise and overlap of the spectrum with other signals.

An alternative is based on the physical interaction sensor system, which aims directly onto the mapping between the human–robot interaction (HRI) and the system state variables. This interaction mainly depends on the impedance delivered from the pilot to the exoskeleton and vice versa [8,9]. Therefore, an impedance interaction model and corresponding force control methodology are desired [10,11]. Moreover, an open-loop impedance controller is designed in [12] for a specific task without feedback derived from the force sensors. An improvement was made in [13], which takes the feedback term into consideration. Nevertheless, the control system could hardly tell apart the HRI and the system disturbance. Thus, there are still substantial uncertainties with respect to model error.

For rehabilitation therapy dealing with the interaction force and a delicate haptic interface, a more accurate impedance dynamic model is required [14]. Thus, several methods of the self-tuning impedance variables are presented in [15] to fulfill the requirement. In addition, a series elastic module has been designed to provide high-fidelity HRI. However, owing to the stochastic human–exoskeleton coupling model, the uncertainties of interaction cannot be avoided.

Apart from the identification process of the interaction model, disturbance observer is a typical technique trick for coping with the dynamic uncertainties, as detailed in [16]. Accordingly, the interaction uncertainty is compensated by the observation from the feedback loop as well as disturbance observer [17]. Nevertheless, a critical limitation of such a controller is that the control performance highly relies on the prior knowledge of the control plant. Besides, in order to obtain an approximation of the interaction torque between the exoskeleton and the user, an independent-joint-based disturbance observer in combination with gravity compensation and friction cancellation is proposed in [18]. Nevertheless, the independent joint-based implementation includes coupled multi-degrees of freedom (DoF) dynamics effects.

To enhance control performance and system stability in the presence of model uncertainties, Kim and Bae propose a novel time-delay robust control algorithm in [19]. Thus, derived from the time-delay design conception, the model uncertainty and the HRI disturbance are compensated according to previous observations. Thus, the desired performance can be maintained since the disturbance and the time-delay problem are solved. However, the parameters of the time-delay term should also be identified, and the corresponding identification process was not well addressed.

An innovation made in [20] is that the approach takes the human model into consideration. In existing passivity-based algorithms, the human model is simplified as a passive system. However, the control performance cannot be guaranteed, owing to the simple human coupling model. In [20], the human model is defined as a second-order dynamic system whose parameters are updated with an adaptive approach. Although the control performance is well-defined, it only works when the system can be given as a second-order environment. Another example of the force control of the series elastic actuator (SEA) takes the effect of load motion compensation into account and includes the excellent work in [21]. Moreover, a force control with acceleration feedback of series elastic actuators is introduced in [22]. Although this method exhibits higher performance accuracy and robustness and is easy to implement, the feasible application is only based on the assumption that the acceleration can be computed analytically or numerically.

In order to provide relatively low output impedance across the entire frequency spectrum, a novel cable-driven actuation for the lower extremity exoskeleton is designed in [23]. Although the performance is sufficient, the output-impedance variations can still be achieved by several closed-loop interaction control methodologies [24]. Moreover, also driven by the flexible Bowden cable transmission, two kinds of series elastic actuators (i.e., linear compression spring and helical torsion spring) are presented in [25,26] for small-scale exoskeleton application; indeed, the linear springs make the mechanical system more bulky over the finger.

Therefore, considering the above limitations, a novel compact elastic module for lower extremity exoskeleton and its corresponding control methodology—so-called evolving internal model control (EIMC)—are designed and proposed in this paper. Based on our previous work [27], the elastic module consists of two parts (i.e., the distal module for compliant control and the proximal module for HRI detection). The crucial drawback of our previous work is that the pilot feels uncomfortable with the proximal elastic module and the mechanical structure is bulky. In terms of the excellent work in [28], a novel high-power series elastic actuator has been proposed. However, the work is mainly designed for rehabilitation purposes. Moreover, the experiments are only tested on a hardware platform. The assistance performance with a human subject is not presented.

**Figure 1:**
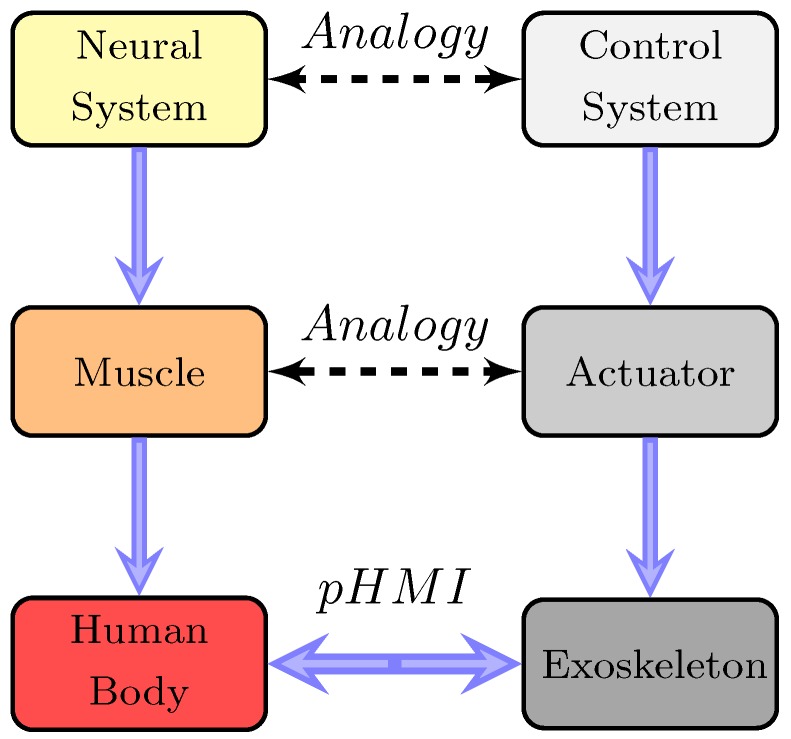
Abstract analogy between the human and the exoskeleton. The control system can be seen as a high-level neural system of the exoskeleton. According to the high-level control signal, the actuator can drive the exoskeleton mechanical structure. Thus, to obtain an input to the controller, the human intention is interpreted from the physical human–machine interaction (pHMI) between the pilot and the exoskeleton.

In this paper, we only apply the distal module to achieve both tasks, and the control concept is given in Figure 1. For enhancing the robustness of the control system, a distributed online learning forward model is introduced. Consequently, the HRI can be seen as the disturbance to the control system, and should be compensated. A crucial feature of our control strategy is that the model uncertainties are addressed by the online learning model and the HRI is approximated by the position error between the output of the forward model and the exoskeleton. However, in most control scenarios, the measured noise, the model uncertainties, and the HRI are all considered as the system disturbance, and cannot be distinguished.

To improve readability, the main contribution is presented as follows:

(1) Based on the intrinsic sensing properties, a novel compact elastic module is designed to provide the input signal for the controller. Moreover, the compact elastic module is also utilized for the compliant actuation.
(2) To improvement the control performance, a novel control scheme—so-called evolving internal model control—is proposed in this paper. The control scheme aims to compensate the system disturbance and model uncertainties by the difference between the exoskeleton control plant and the forward learning model.
(3) In order to enhance the system robustness, distributed online model learning is introduced in this paper. Additionally, the main issue of the computation expense is addressed with the distributed learning framework, and the hyper-parameters for each Gaussian process are updated with the Markov Chain Monte Carlo (MCMC) algorithm.
(4) Finally, to demonstrate our control scheme, the model learning procedure as well as the system properties are tested on our exoskeleton robotic system with several experiments.

The remainder of the paper is organized as follows:

After the introduction, Section 2 is devoted to discussing the intrinsic sensing and the mechanical design of the compact elastic module in light of the biomechanical inspiration. In Section 3, the main routine of the EIMC scheme is outlined, along with the discussion of the distributed evolving model learning. The implementation of the experiments and the comparison of the control methods are presented in Section 4. Finally, Section 5 concludes with a summary.

## 2. Compact Elastic Module

In few exoskeleton design scenarios, the mechanical structure is designed as a rigid body robotic system and the biomechanical aspect may not been well considered. Consequently, the wearer may feel uncomfortable since the natural movement is constrained. In this section, we first discuss the motion range and actuation from the point view of the biomechanical inspiration. Then, the design of a compact elastic module is introduced, and the corresponding sensing properties are carefully explained.

### 2.1. From the Biomechanical Inspiration

Human biomechanics should be taken into consideration when designing an exoskeleton robot, “since human legs have inherent damping as well as stiffness properties” [29]. In almost all daily human life, the leg joints can be simplified as damped spring and mass—especially for sitting, standing up and walking. The viscous damping is of great importance in the functioning of movement primitives. Such a property plays a significant role in shock absorbing as well as maintaining stability.

Towards the aim of forming a mechanical scheme of an exoskeleton, all the design layouts almost fall into three classical types; i.e., anthropomorphic, non-anthropomorphic, and pseudo-anthropomorphic. An anthropomorphic scheme seeks to match all the human body physical properties precisely (i.e., limb length, joint range, and misalignment). Nevertheless, in practice, scarcely a perfect anthropomorphic system can be designed regarding critical requirements ranging from exact end-effector matching as well as joint misalignment. Besides, although non-anthropomorphic design concepts are widely used in many scenarios, it is quite difficult to fulfill all the possible maneuvers (deep squats and turning corners) for ankle joints.

Therefore, we consider the above limits and design our exoskeleton based on pseudo-anthropomorphic architecture. This means that the developed mechanical scheme is kinematically similar to the human biomechanics; i.e., three DoFs at both ankle and hip joints, one DoF for the knee joint. Moreover, at the very least, the range of each joint should cover the range of human locomotion and less than the maximum range of human motion for safety concerns. Thus, the whole range of every joint is given in Table 1.

**Figure 2:**
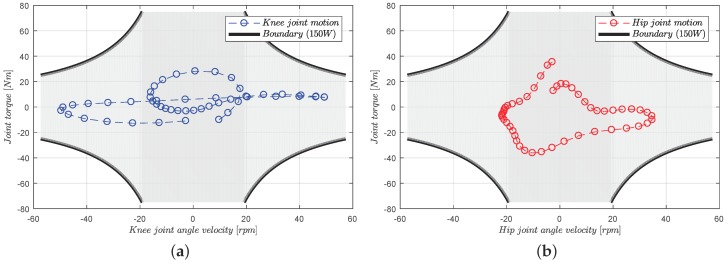
Provement of electric motor selection. For electric actuation, the power of the selected electric motors should cover the range of the joint torque during normal human walking. For safety concerns and efficiency, we chose the RE40 dc motor (150 W) from the Maxon Motor Company. (**a**) Motion range of the knee joint; (**b**) motion range of the hip joint.

**Figure 3:**
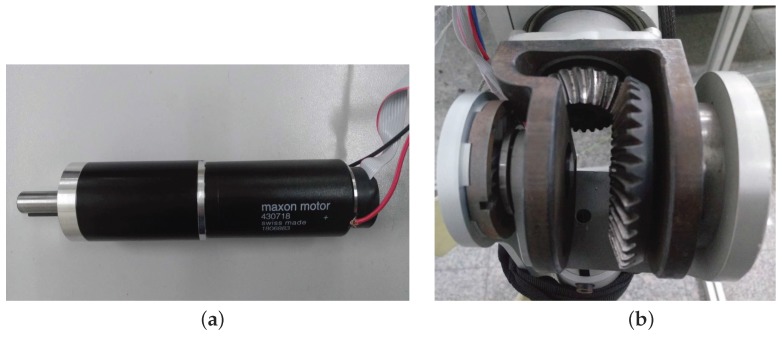
Mechanism of joint power transmission. Considering the scope of the joint torque and compactness of the mechanism, we chose the above scenario (i.e., electric motor with spur gearhead and bevel gears). (**a**) Electric motor RE40 and spur gearhead (26:1); (**b**) bevel gears (4:1).

To fulfill the requirement of normal human walking [30,31] assistance, the exoskeleton robotic system should provide a large range of torques, and the selection of the proper electric motors should be well considered since the maximum speed and torque are limited (presented in Figure 2). In our situation, since the maximum continuous speed and torque of the selected motor (RE40, Maxon Motor Company, Sachseln, Switzerland) [32] are 8200 rpm and 0.181 Nm, the corresponding parameters should be designed in proper scope. Thus, a spur gearhead (26:1) and a pair of bevel gears (4:1) (given in Figure 3) are applied to adjust the speed and torque to 78.8 rpm and 18.8 Nm, separately. The maximum angular speed is near 60 rpm for the strength augmentation, and the exoskeleton should be capable of tracking the human primitives in real-time. Therefore, the maximum driven torque (268 Nm) is large enough for the exoskeleton actuation plus the stall torque (249 Nm).

### 2.2. Designing and Sensing of a Compact Elastic Module

**Figure 4:**
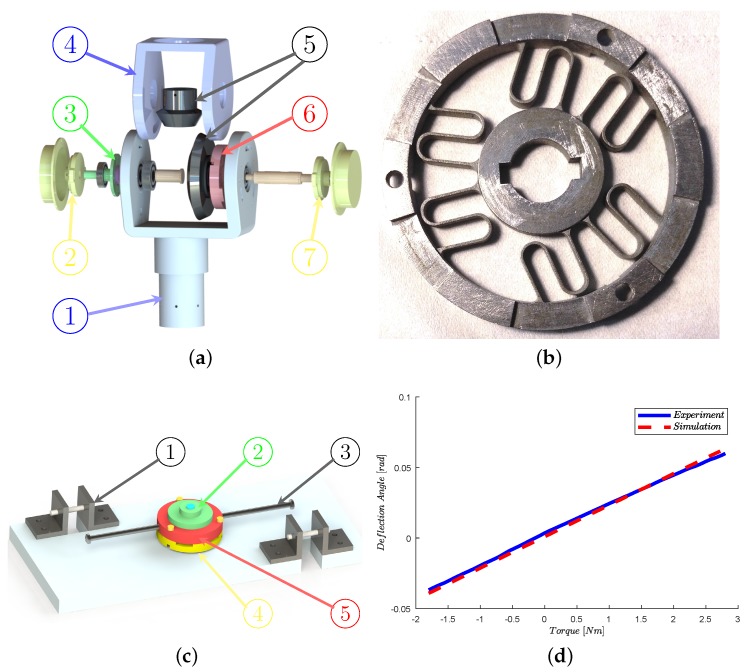
(**a**) Configuration of the compact elastic module; 1: Shank segment, the torsional elastic module, 2: Encoder, 3: Encoder support, 4: Thigh segment, 5: Bevel gear pair, 6: Torsional elastic module, and 7: Encoder. (**b**) Front view of the torsional elastic module; properties of the designed torsional elastic module are given in Table 2. (**c**) The calibration platform; 1: Bracket, 2: Encoder, 3: Force-bearing bar, 4: Torsional elastic module, 5: Position pin. (**d**) Calibration result of the elastic module; experimental data is presented in the solid blue line, while the simulation data are given in the red dashed line.

In our previous work [27], two elastic modules were applied for compliant control and human intention recognition, separately. In this paper, we only use one elastic module and combine the intrinsic sensing property of the elastic module and the compliant actuation to improve the compactness of mechanical design, presented in Figure 4a. The deflection of the elastic module is measured through the right encoder, while the incremental joint position is obtained by the left encoder. Consequently, the input to the controller is interpreted as the deflection of the elastic module. Moreover, the HRI force is compensated by our proposed EIMC and will be carefully explained in the next section.

Since the estimation of human motion recognition highly depends on the accuracy and stability of the physical HRI, the elastomer is of great significance in the sensor design. The common options of topology structures are symmetrical and centrosymmetric. The main drawbacks of the symmetrical structures are higher processing costs and less optimized parameters. Based on the analysis in [33], the architecture of spiral and double helix are usually neglected. Additionally, the linearity and the stiffness cannot be guaranteed, owing to the backlash. Therefore, we design a centrosymmetric structure elastic module, as shown in Figure 4b. With the parallelogram-like deflection between the inner circle and outer circle, the human motion is measured by the deflection of the elastic module through the encoder. Moreover, the designed parameters are given in Table 2.

A calibration procedure is required for sensing in a robotic system, and a calibration platform was designed as presented in Figure 4c. The body of the elastic module is grounded on the platform, and the deflection is measured with the encoder mounted on the outer ring. Additionally, the external torque is generated by the force-bearing bar. The measurement signal is obtained from a programmable multi-axis controller (PMAC, Delta, Chatsworth, CA, USA). During the calibration experiment, the loading cell straining on the elastic module ranges from 100 g to 2.5 kg with 50 g increments. The results given in Figure 4d show that the stiffness is Ke=60.2 Nm/rad. Compared with simulation, the calculation error is 4.5%. Moreover, the resolution 0.1 Nm is guaranteed by the 12-bit resolution magnetic encoder.

## 3. Evolving Internal Model Control

The internal model control [34,35] is one of the standard applied model-based techniques for nonlinear control systems. However, for lower extremity exoskeleton systems, the dynamic model is not easy to obtain. Although several black-box modeling methods [36,37] have been combined with internal model control for dynamic model identification, the barriers are mainly associated with the curse of dimensionality. In this section, we briefly introduce the essential properties of internal model control and carefully address the modeling issue with our proposed online learning algorithm.

### 3.1. Internal Model Control with Gaussian Process

For realizing ideal motor control of an exoskeleton which interacts with a pilot, the joint friction, HRI, and inertia should be compensated. Compared with the internal model control, the primary limits of the classical control theory fail to explicitly model the dynamic system according to a classical feedback loop. Thus, to enhance the control performance, internal model control is employed in this paper. In [38], the forward model is learned with a full Gaussian process. The prediction of the Gaussian process provides mean as well as variance. This variance can be seen as the level of confidence of the prediction, which is a crucial advantage compared with neural network or fuzzy models. Note also that the variance is explicitly applied when the data-stream subset should be updated (remove data pair or add data pair). Moreover, owing to the Gaussian process theory, each time step prediction is approximated as a Gaussian process, which is determined by the mean and the variance.

**Figure 5:**
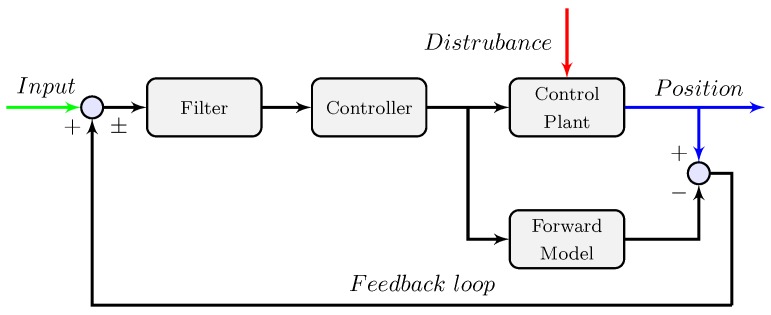
A general representation of the internal model control. The design concept of the internal model control is to compensate for errors or make adjustments from the desired outputs. Thus, two primary parts of the control algorithm are the forward model and feedback loop used to enhance the control performance.

As shown in Figure 5, the reference input to the controller is related to the desired position. The motor command generated by the controller is sent to the control plant and a forward model. Thus, compared with the output from the control plant, disturbance from the environment may cause differences between the predicted positions and the actual system states. The designed filter balances the robustness to the model uncertainties and the desired closed-loop behavior of the control system.

Compared with the other state-of-the-art control algorithms, the crucial advantages consist of the following properties.

**Property** **1** (Dual Stability Criterion). If the model is accurate, the stability of both plant and controller is sufficient for overall system stability.

**Property** **2** (Perfect Controller). If the controller can be achieved as the inverse of the model, the perfect controller can be obtained.

Since the exoskeleton is a typical robotic system with high coupling and inherent nonlinearity, we apply adaptive computed torque control to address this issue. Thus, without loss of generality, the driven torque is given as:

(1) $$\tau =\hat{H}\left(\theta \right)\left({\ddot{\theta }}_{d}+K_{d}\dot{e}+K_{p}e\right)+\hat{C}\left(\theta ,\dot{\theta }\right)\dot{\theta }+\hat{G}\left(\theta \right),$$

where $\hat{H}$, $\hat{C}$, and $\hat{G}$ denote the estimations of the inertial matrix, the centrifugal and Coriolis term, and the gravitational matrix, respectively. Considering the real exoskeleton dynamic model, we have

(2) $$H(\theta )\ddot{\theta }+C(\theta ,\dot{\theta })\dot{\theta }+G(\theta )=\hat{H}\left(\theta \right)\left({\ddot{\theta }}_{d}+K_{d}\dot{e}+K_{p}e\right)+\hat{C}\left(\theta ,\dot{\theta }\right)\dot{\theta }+\hat{G}\left(\theta \right),$$

where H, C, and G denote the inertial matrix, the centrifugal and Coriolis term, and the gravitational matrix, respectively. In light of the linear connection of the inertial parameters, we have

(3) $$\begin{array}{cc} \hat{H}\left(\theta \right)\left(\ddot{e}+K_{d}\dot{e}+K_{p}e\right) & =\tilde{H}\left(\theta \right)\ddot{\theta }+\tilde{C}\left(\theta ,\dot{\theta }\right)\dot{\theta }+\tilde{G}\left(\theta \right) \end{array}$$

(4) $$\begin{array}{c} =Y(\theta ,\dot{\theta },\ddot{\theta })p, \end{array}$$

with $\tilde{H}≜H-\hat{H}$, $\tilde{C}≜C-\hat{C}$, and $\tilde{G}≜G-\hat{G}$. Note that only p includes all the inertial parameters. To demonstrate the stability, we transform the equation into the following form:

(5) $$\dot{x}=\left[\begin{array}{cc} 0 & I\\ -K_{p} & -K_{d} \end{array}\right]x+\left[\begin{array}{c} 0\\ -I \end{array}\right]\Phi p≜Ax+Bp,$$

with $x={\left[e^{T},{\dot{e}}^{T}\right]}^{T}$ and $\Phi ={\tilde{H}}^{-1}Y$. Therefore, the control system is stable, since both $K_{p}$ and $K_{d}$ are defined as positive definite matrix. Moreover, p is evolved according to $\dot{p}=\Gamma^{-1}\Phi^{T}B^{T}Rx$, where Γ is also denoted as a positive definite matrix. Besides, the following Lyapunov equation should be satisfied and R is the only solution:

(6) $$A^{T}R+RA=-Q.$$

Since the control system is stable, the first property is easily satisfied. Regarding the second property, the perfect controller which can be designed as the inverse of the dynamic model is quite difficult to obtain, owing to HRI disturbance and model uncertainties. However, if the accurate model can be achieved, the disturbance can be compensated to the next time step control input. Therefore, the precision of the robotic exoskeleton system can be enhanced.

### 3.2. Offline Distributed Learning of Forward Model

Although the Gaussian Process is widely applied in many robotics programs, the computation expense is a strong barrier to the implementation of online model learning. The distributed forward model learning proposed in this paper aims to lower the computational burden by explicitly applying several subsets of the training data. In the past five years, the sparse Gaussian process has been widely used in spatiotemporal modeling [39] as well as robotics reinforcement learning [40]. However, it is inconceivable to use sparse approximations with a data set size of $N\approx O(10^{7})$ [41]. To further reduce computation expense, we employ distributed computations as an alternative method. In [41], the application of the distributed Gaussian process for deterministic test point has been validated. In this paper, we extend for a more complicated task (i.e., the distributed Gaussian process for test point with a distribution).

“A Gaussian process is a collection of random variables, any finite number of which have a joint Gaussian distribution” [42]. Consider a regression problem y=f(x)+ϵ∈R, where $x\in R^{D}$. The likelihood $p\left(y|f\left(x\right)\right)=N\left(f\left(x\right),\sigma_{\epsilon }^{2}\right)$ explains for the i.i.d. measurement noise $\epsilon \in N(0,\sigma_{\epsilon }^{2})$.

The states of the system are joint position q and angular velocities $\dot{q}$. Moreover, the control signal u<40 Nm is constrained for safety concerns and selected with a random value for training. Thus, the forward model is implemented as a Gaussian regression, where the training inputs are defined as tuples $x_{i}^{\left(t\right)}$ = ($q_{t}$, ${\dot{q}}_{t}$, $u_{t}$) and differences $y_{i}^{\left(t\right)}$ = $\Delta q_{t}=q_{t+1}-q_{t}$ as training target, with i=1,…,T. Throughout this paper, the squared exponential kernel *k* with automatic relevance determination is given as

(7) $$k\left(x_{i}^{\left(t\right)},\left(x_{j}^{\left(t\right)}\right)\right)=\alpha^{2}\exp\left(-\frac{1}{2}{\left(x_{i}^{\left(t\right)}-x_{j}^{\left(t\right)}\right)}^{T}\Lambda^{-1}\left(x_{i}^{\left(t\right)}-x_{j}^{\left(t\right)}\right)\right),$$

where $\theta =\{\Lambda ,\alpha^{2}\}$ (length-scales vector Λ, signal variance $\alpha^{2}$).

Under the distributed framework, we assume that the *M* Gaussian processes (GPs) are independent. Thus, the marginal likelihood p(y|X,θ) for a full GP training set can be decomposed into the product of *M* individual parts

(8) $$p\left(y|X,\theta \right)=\prod_{k=1}^{M}p_{k}\left(y^{\left(k\right)}|X^{\left(k\right)},\theta \right),$$

where each term $p_{k}$ is characterized by the *k*th GP. In addition, we share a set of hyper-parameters θ for all the *k* GPs. To optimize the hyper-parameter θ, a common method is maximized with the evidence maximization. However, in this paper, we employ more efficient method (i.e., expectation maximization with Monte Carlo sampling).

Additionally, the expectation step aims to compute the posterior distribution of the states $q\left(X\right)=p\left(X^{\left(k\right)}|y^{\left(k\right)},\theta \right)$. Since the posterior distribution is not GP and is difficult to compute analytically, we draw several samples from the posterior distribution according to Monte Carlo sampling. Consequently, the true distribution which is not a GP is approximated by a GP distribution. Thus, the marginal likelihood can be given by Monte Carlo integration. For the maximization step, the hyper-parameters are obtained from maximizing the expected likelihood $E_{q}\left[p\left(X^{\left(k\right)}|y^{\left(k\right)},\theta \right)\right]$ with stochastic conjugate gradient. The main routine is given in Algorithm 1. Therefore, the learning procedure is implemented with 10 multiprogramming (GP C++ library [43]) under a distributed framework. Note that the data is collected with proportional derivative (PD) control on the above-described system, and each data volume contains 1000 data pairs.

**Figure 6:**
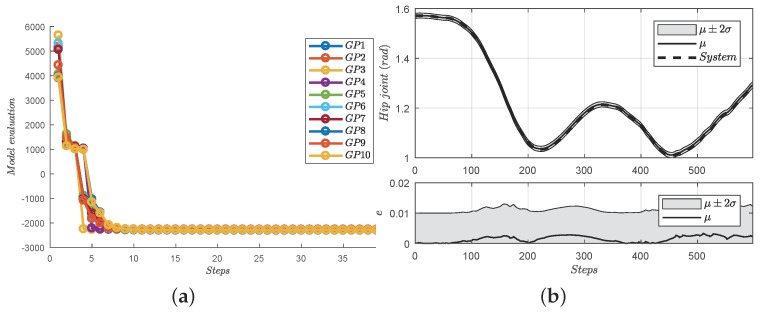
Learning procedure and cross-validation of the distributed forward model. (**a**) Learning procedure hyper-parameters of 10 GPs; (**b**) cross-validation of learned forward model. The efficient training process has been explained in the above figure. In addition, since the trust region of the trained model is about 0.57 deg, the precision for the practical experiment can be accepted.

**Figure 7:**
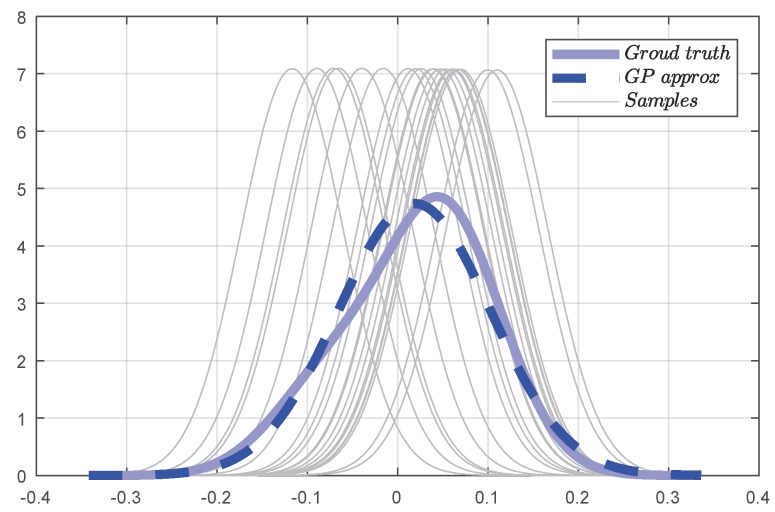
Graph interpretation of the Monte Carlo sampling. Note that the true distribution is not a GP as given with a dashed blue line, and it is evaluated as a GP shown in the solid blue line with 20 samples.

As can be seen in Figure 6a, the learning procedure requires about 30–40 trials each GP. Since the evolution function is initialized with a random condition, the first step penalty may be different. Moreover, during the training, the graphic interpretation of Monte Carlo sampling is given in Figure 7. Consequently, with 20 samples, the distribution can be approximated by a GP distribution. In order to verify the distributed learning forward model, the 600 unused data pairs are tested with the model, given in Figure 6. In addition, as can be seen in the Figure 6, the hip joint model propagates the uncertain input (with system noise) to a next-step output joint position with nearly 0.01 rad in the learning region of interest.

**Algorithm 1:** Calculate Hyper-Parameters Value with Monte Carlo Expectation Maximization **input**: Training data $X^{\left(k\right)}$, $y^{\left(k\right)}$, where k∈1,…,M; **output**: Hyper-parameters θ; 
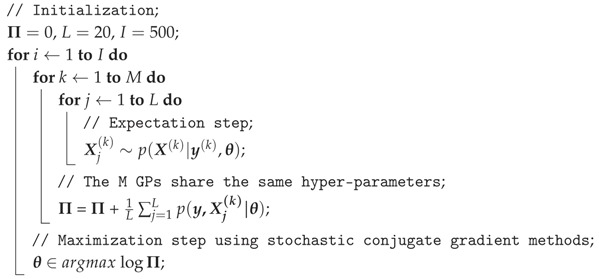


To combine the *M* GPs to obtain a whole prediction, we compare four fusion algorithms; i.e., product-of-GP-experts (PoE) [44], generalized PoE (gPoE) [45], the Bayesian committee machine (BCM) [46], and the robust Bayesian committee machine (rBCM) [41]. To choose a fusion method, we tested one dimension problem [47] with the four algorithms.

**Figure 8:**
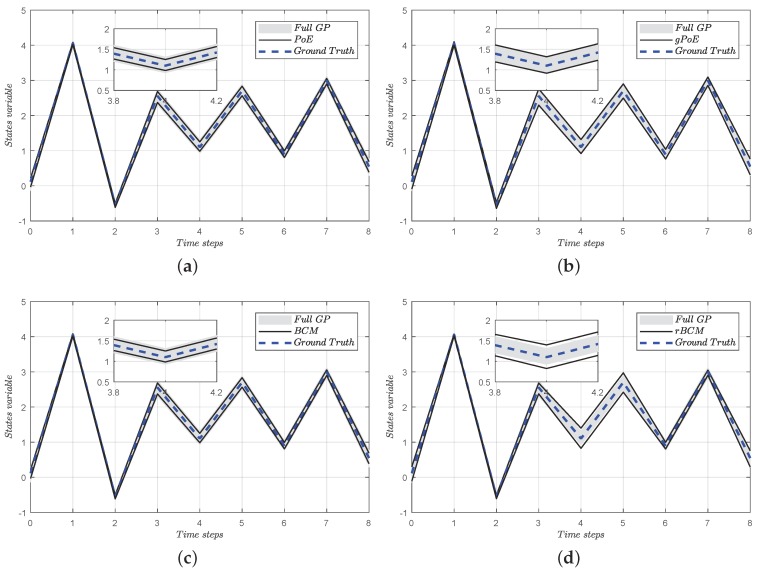
Comparison of four data fusion methods (product-of-GP-experts (PoE), generalized PoE (gPoE), Bayesian committee machine (BCM), robust Bayesian committee machine (rBCM)) for the distributed forward model. In each subfigure, the confidence interval of the comparison algorithm is filled between two black lines. Additionally, the full GP to be approximated is shown in the gray shaded area, representing 95% of the confidence interval, and the ground truth is given in blue dashed line. We apply NLL (negative log-likelihood) to evaluate the fusion performance presented in Table 3. (**a**) PoE; (**b**) gPoE; (**c**) BCM; (**d**) rBCM.

As shown in Figure 8 and Table 3, compared to the full GP prediction (negative log-likelihood (NLL): −3.12), both PoE (NLL: −3.22) and BCM (NLL: −3.25) overestimate the variance in the region of interest and result in overconfident precision. Although a more robust solution is desired, the predictive result of the rBCM (NLL: −2.54) is too conservative. On the contrary, the gPoE (NLL: −2.81) provide a more reasonable prediction mean and variance.

Therefore, considering the above analysis, the gPoE is applied in a distributed inference framework as shown in Algorithm 2. Moreover, propagating through a nonlinear system, the output is not a GP model. Thus, the output distribution can be seen as a Gaussian mixture and evaluated by Monte Carlo approach.

**Algorithm 2:** Inference with Markov Chain Monte Carlo **input**: Training data $D=X^{\left(k\right)}$, $y^{\left(k\right)}$, where k∈1,…,M, Hyper-parameters θ; **output**: The inference Mean $\mu_{*}$ and Variance $\sigma_{*}$; 
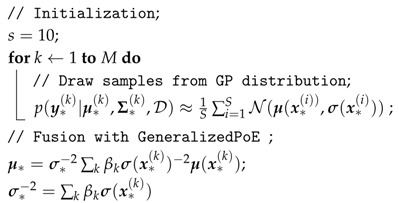


### 3.3. Online Distributed Evolving of Forward Model

In this subsection, we seek to present an online model learning algorithm. Such an algorithm is desired since the training data collected under the implementation of another control scheme is not entirely precise or when the dynamics model is time-varying. In addition, although the reinforcement learning is applied to several robotic cases [48,49], leaning an exoskeleton dynamic model with human interaction is not recommended due to safety concerns.

For online learning implementation, the critical barrier is computation expense. Based on our distributed prediction scheme in Section 3.2 and inspired by the interesting work in [50], we propose a distributed online evolving model learning methodology, and its main routine is given in Algorithm 3.

**Algorithm 3:** Distributed Online Model Evolving **input**: Observation ($z_{*},y\left(z_{*}\right)$), predictive distribution $N(\mu \left(z_{*}\right),\sigma^{2}\left(z_{*}\right))$; **output**: Updated hyper-parameters $\theta^{new}$; 
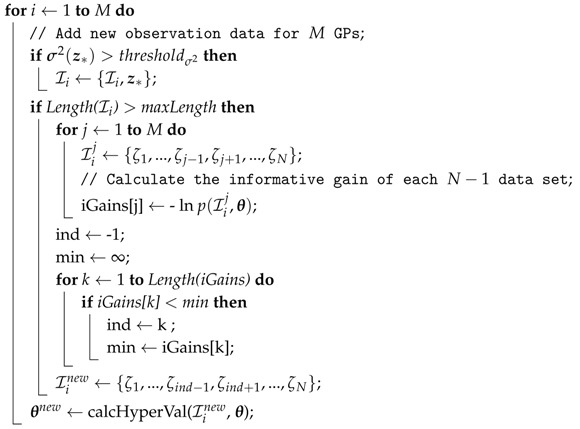


The general concept of the distributed evolving model can be explained in the following steps: first, for *M* initial data sets with trained hyper-parameters, the new observations $z_{*}$, such as sensor data derived from encoder, inertial measurement unit (IMU), drivers, and deflection of compact module are added to difference *M* sets $I_{i}$ for next step evaluation; second, calculate each *j* informative gain using iGains[j] data sets $I_{i}^{j}$ with data *j*, where j=1,…,N; according to the informative gains, find the observation with the worst information gains and remove it to form a new data set $I_{i}^{new}$; finally, the new hyper-parameter $I_{i}^{new}$ should be recalculated in terms of $I_{i}$ as well as θ, and the updated covariance K is obtained. The procedure is repeated for every incoming loop until a terminate command is received or there is no more available data.

Not akin to the algorithm in [50], the judge condition of adding a new observation only depends on the prediction variance. To be more specific, if the prediction variance is higher than the threshold value, it can be seen that the model is not confident, even though the difference between the predictive mean and its threshold is small enough. Moreover, the hyper-parameters are calculated by the sum of the marginal likelihood of the *M* GPs according to Algorithm 1.

**Figure 9:**
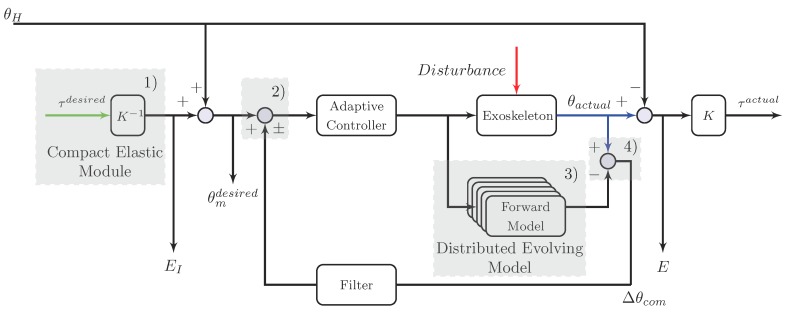
General representation of the evolving internal model control.

Therefore, the whole control scheme as shown in Figure 9 can be introduced as follows.

(1) First, the desired position $\theta_{m}^{desired}$ can be obtained from the deflection $E_{I}$ of the elastic module. If the pilot keeps a steady pose, there is no incremental difference between the motor position and the human joint angle. Therefore, the desired position $\theta_{m}^{desired}$ is zero.
(2) Second, the input to the adaptive controller consists of the desired position $\theta_{m}^{desired}$ as well as the error position compensation $\Delta \theta_{com}$ from the feedback loop.
(3) Then, the same torque command is sent to the distributed online model and the exoskeleton system with the signal amplification by the driver. Moreover, the *M* new observation pairs are evaluated with Algorithm 3 and the additional data will be added to the new *M* subsets if the condition is satisfied.
(4) Finally, based on the internal model control framework, the error position $\Delta \theta_{com}$ is compensated through the feedback loop. Besides, the low-pass filter is applied to enhance the robustness of the control system.

**Online Computation Expense:** The main issue of the Gaussian process online learning algorithm is the computation cost. As summarized in Table 4, if *N* is defined as the data size, the training and prediction of the full Gaussian process scale in $O(N^{3})$ and $O(N^{2})$. This inherent weakness limits the practical application. In terms of the sparse Gaussian process, the computation burden is lowered by implicitly or explicitly using a subset of the data (*Q* ≈ N/10). Nevertheless, it is impossible to apply Gaussian process to a training set size of ($N\geq O(N^{7})$). Hence, to further improve the computation efficiency, “the distributed Gaussian process can handle arbitrary large data sets” with sufficient hierarchical product-of-expert model [41], which is used in our offline model learning. Since we cannot capture all the possible scenarios, the distributed online model evolving is employed in this paper. Thus, under the distributed framework, computing the inverse covariance of the incoming data stream for *M* subsets scales in $O(MP^{3})$ or $O(MP^{2})$ for covariance updating or only subset changing in light of [50]. Concerning the inference, our proposed online algorithm scales in $O(MP^{2})$.

## 4. Experiment

Although the control system for the compact elastic module is designed and explained in the previous part, it is crucial to verify the performance of several experiments. The capabilities of the control system (e.g., the control precision, disturbance rejection, and minimal impedance and back drivability with the human subject) will be demonstrated in the following.

### 4.1. Hardware Configuration

**Figure 10:**
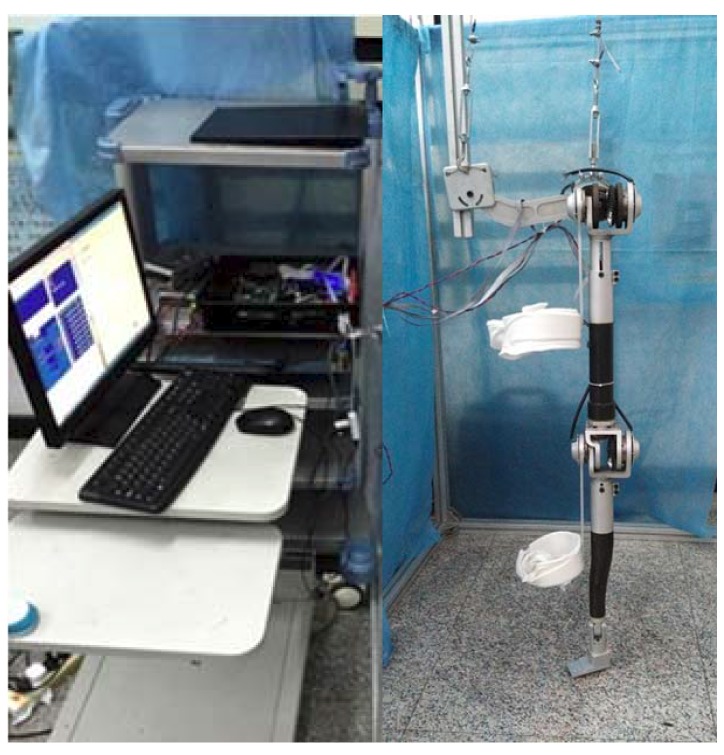
Exoskeleton robotics system. The whole control system consists of the embedded PC, programmable multi-axis controller (PMAC), Copley actuators, power supplement, and required auxiliary facilities integrated into the control enclosure.

The following experiments are verified on our single-leg exoskeleton robotic platform, as shown in Figure 10. The platform is designed as an ergonomic system for strength augmentation as well as endurance enhancement. The motion range for each joint has been carefully explained in Section 2, along with the mechanical structure of actuation with the compact elastic module.

For our situation, three kinds of voltage (i.e., 5 V, 12 V, and 24 V) are desired. The overall control scheme is written in embedded PC, and the driven commands are sent to the electric motors through Copley driver. Moreover, the PMAC (programmable multi-axis controller, Delta, Chatsworth, CA, USA) is utilized for motion planning and signal collection.

### 4.2. Target Tasks

This experiment specifically targets three robot-aided tasks.

#### 4.2.1. System Properties

Since the additive part of the input to the controller is derived from the feedback control loop, a low-pass filter is desired to enhance the system robustness. Therefore, the frequency scope of the designed filter should at least cover the range of bandwidth of the human motion. Moreover, the filter should not be sensitive to the system noise in the high-frequency domain. Consequently, the candidates of our filter have the following form: $F\left(s\right)=\frac{\sum_{k=0}^{M}{\left(\tau s\right)}^{k}}{{\left(\tau s+1\right)}^{N}}$ [52,53]. In our case, the *N* and *M* are set to be 3 and 1. Moreover, the uncertainties Δ are the difference of the exoskeleton plant between stance and swing phase [17].

The system bandwidth is a crucial parameter to evaluate the control performance of the system, since the frequency components of the human motion are up to 4–8 Hz, as detailed in [54]. To respond to the control signal quickly, the bandwidth should at least cover the frequency components of the human motion. In this task, the experimental preparation is the same as detailed previously. Both PD algorithm and our proposed method were tested on our exoskeleton system without a human subject.

To evaluate the control precision, we conducted a simple experiment with specified position signals on the same exoskeleton system. The first-period signal was the zero pose command; the knee joint of the exoskeleton was asked to keep a steady position to evaluate the control performance with existing static friction force and bias force. The second period was to control the exoskeleton with sinuous-like position command to test the control performance against the model uncertainties. The compared algorithm was also a classic PD control scheme.

#### 4.2.2. Algorithm Comparison with Human Subject

**Figure 11:**
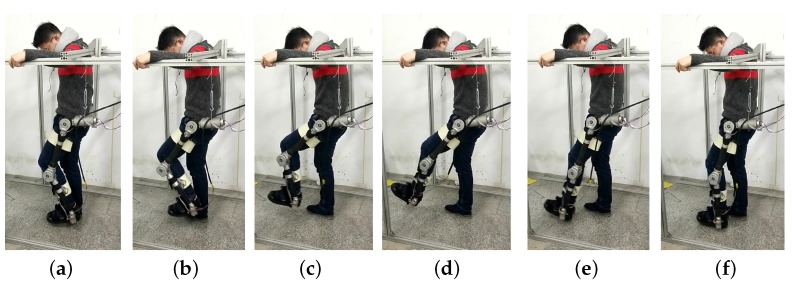
Experiment with the human subject (subject A) during the swing phase of normal locomotion. (**a**) Heel rise; (**b**) initial swing ; (**c**) middle swing; (**d**) terminal swing; (**e**) heel strike; (**f**) full ground contact. With the compliant interaction between the user and exoskeleton, the human subject was asked to sway his left leg naturally. The human intention is recognized by the deflection of the elastic modules mounted on both hip and knee joints.

To verify the output impedance and back drivability of the control system with the compact elastic module, the sway experiment with a human subject (subject A) is presented in this task. The human subject was asked to sway his leg naturally in the experiment, as shown in Figure 11. Note that the HRI was made possible with the compliant cuff.

The compared state-of-the-art algorithms are derived from [17,33]. The reason for choosing these two algorithms for comparison is because the experimental hardware is also a single-leg exoskeleton system with series elastic actuators and the algorithms are also based on the compensation of the human interaction, which is considered as the disturbance of the control system. However, the core idea of such algorithms is disturbance observer. Since the control performance for the exoskeleton robot is difficult to verify, we follow the same evaluation tool (i.e., root-mean-square error (RMSE)) used in [17,33] to test the effectiveness of our proposed method.

#### 4.2.3. Experiment with Different Individuals

Although we aim to provide a control scheme for any human subject since the forward model is first learned offline and designed to be evolved online, the control performance should be verified with different human subjects. Thus, the human subjects’ information is briefly given in Table 5. Similar to the comparison task, the human subjects were asked to naturally to sway their leg, and the system hardware was also the same as detailed above.

### 4.3. Experimental Results and Discussion

#### 4.3.1. System Properties Results

**Figure 12:**
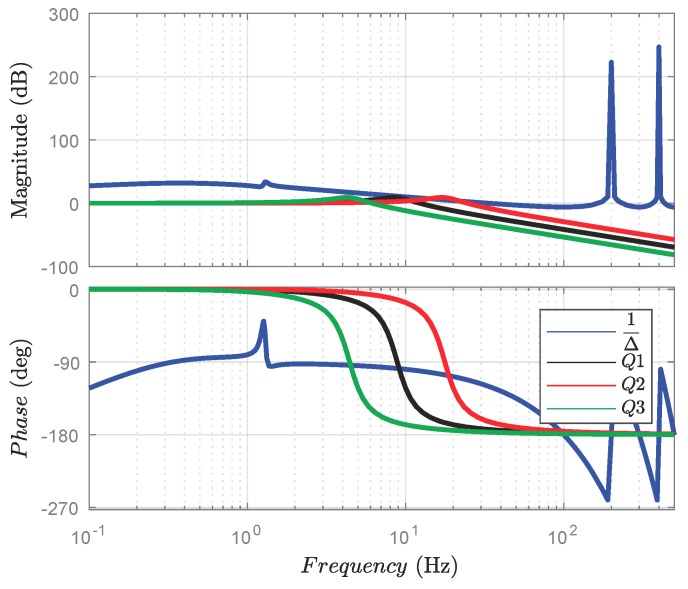
The design of the feedback-loop filter. Since the filter is employed to enhance the closed-loop robustness, the frequency scope of the designed filter should cover the range of bandwidth of the human motion. Moreover, the filter is supposed to remain below and closed to the uncertainties 1/Δ.

In terms of the enhancement of the closed-loop robustness, the filter was designed to eliminate high-frequency disturbance. The main issue for such a filter is the balance between the robustness against the disturbance and the disturbance rejection. If the frequency range of the filter is too extensive, the system noise poses a threat to the stability. If the frequency range of the filter is too narrow, the disturbance cannot be well compensated. The three candidate filters with the time constants 0.15, 0.075, and 0.030 are given in Figure 12. Since the magnitude response of the filter should remain below and closed to the uncertainty 1/Δ. Hence, the filter with the time constant 0.15 (in solid black line) was chosen.

**Figure 13:**
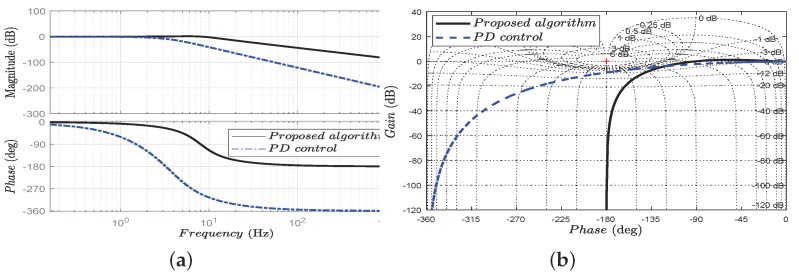
The Bode diagram and the Nichols chart of the control system. (**a**) Frequency response for different control scheme; (**b**) nichols chart for different control schemes. For verifying the open-loop stability, the Bode diagram and Nichols chart are presented with the PD control algorithm and our proposed algorithm. Although both algorithms are stable in the open-loop, the bandwidth of our proposed algorithm is more extensive and hence responds more quickly. In addition, the control system with the PD controller may lose information of the normal high-frequency motion.

The experimental results as presented in Figure 13a show that the system can generate the desired input up to nearly 11 Hz, which for actuation with the compact elastic module is sufficient. However, the bandwidth of the PD controller is only 4 Hz, and the control performance may be “conservative”. The rest information of our algorithm given in the Nichols Chart in Figure 13b is the phase margin (89.99 deg), gain margin (100 dB), and peak magnification (1.2 dB). Therefore, the open-loop control system is stable.

**Figure 14:**
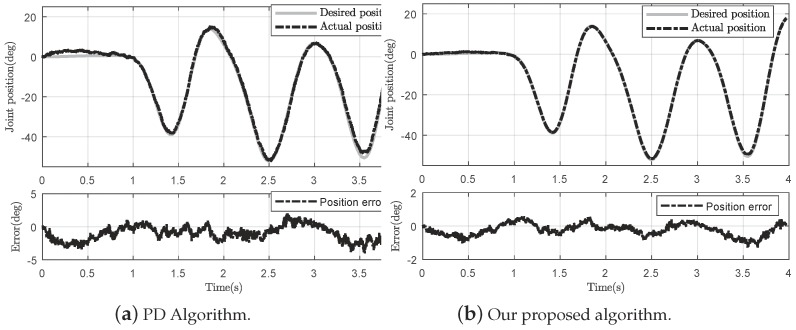
Comparison of the PD control method and our proposed algorithm with the same planning trajectory without human factor.

In order to test the control precision, the experiment implemented in Figure 14 compares the PD control method with our proposed algorithm. Since the static friction effect and bias force exist all the time, they pose a threat to the control precision. As shown in Figure 14a, the PD controller is strongly influenced by the friction effect or the measurement noise—even if the input to the controller is zero position. However, the same situation is better with our proposed algorithm, owing to disturbance compensation and system error adjustment. Moreover, a tracking error occurs almost at time 3.5 s with $4^{\circ }$ in Figure 14a, while at the same high jerk the error is less than $2^{\circ }$ in Figure 14b. Consequently, the RSME of the PD control is 5.78%, while the RMSE of the proposed control scheme is 2.07%.

#### 4.3.2. Results of the Algorithm Comparison with Human Subject

**Figure 15:**
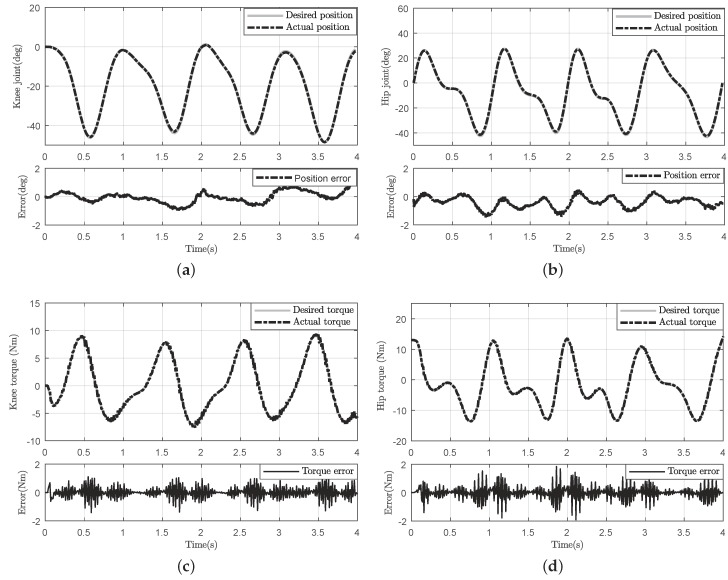
Experiment with a human subject. (**a**) Knee joint tracking and corresponding tracking error; (**b**) hip joint tracking and corresponding tracking error; (**c**) desired torque and actual torque of knee joint; (**d**) desired torque and actual torque of hip joint.

Since human locomotion is not always the same, the trajectory given in Figure 15 may be slightly different. Additionally, since our task is to follow the human motion in real-time, the exoskeleton tries to track the joint motion according to the deflection of the elastic module measured by the encoder. Therefore, in both hip and knee trajectories, the error occurs mostly in the high jerk, as shown in Figure 15a,b. To quantitatively verify the control precision, the RMSE was computed for hip joint 3.26% and knee joint 2.89%, respectively.

Moreover, in order to actuate the exoskeleton effectively and interpret the human intention precisely, the resistive torque should be minimized. Consequently, the human subject will feel more comfortable and natural during the experiment. Thus, the desired torque and actual torque of both joints are given in Figure 15c,d. Additionally, the RMSE for hip joint and knee joint were 4.36% and 4.73%, respectively. Additionally, compared with the other algorithms, the RMSEs of the torque error of the knee joint were 5.5% [17] and 9% [33], as given in Table 6. Thus, our control scheme can provide more precise driven torque and may be more suitable for an exoskeleton application.

#### 4.3.3. Experimental Results with Different Individuals

**Figure 16:**
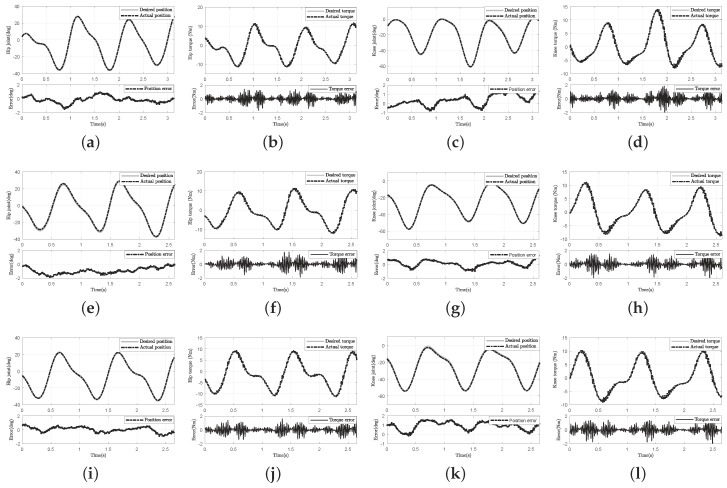
Experiment with the other three human subjects during the swing phase of normal locomotion. The results of three human subjects (B, C, D) are presented in order from the first row to the third row. (**a**) Hip joint tracking and corresponding tracking error of human subject B; (**b**) desired torque and actual torque of hip joint of human subject B; (**c**) knee joint tracking and corresponding tracking error of human subject B; (**d**) desired torque and actual torque of knee joint of human subject B; (**e**) hip joint tracking and corresponding tracking error of human subject C; (**f**) desired torque and actual torque of hip joint of human subject C; (**g**) knee joint tracking and corresponding tracking error of human subject C; (**h**) desired torque and actual torque of knee joint of human subject C; (**i**) hip joint tracking and corresponding tracking error of human subject D; (**j**) desired torque and actual torque of hip joint of human subject D; (**k**) knee joint tracking and corresponding tracking error of human subject D; (**l**) desired torque and actual torque of knee joint of human subject D.

**Figure 17:**
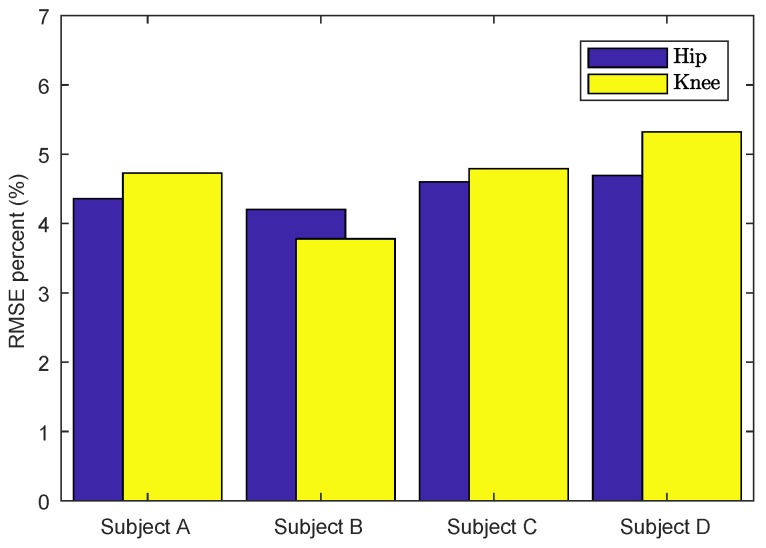
RMSE comparison of torque error for four human subjects.

The experimental results of different individuals are reported in Figure 16 and Figure 17. Since the forward model is learned for the exoskeleton dynamic model and evolved online, the control scheme should be efficient for any human subject. Thus, we added three volunteers’ (subjects B, C, and D) experimental results in Figure 16. Comparing the eight figures of the different joint motion of different individuals (i.e., Figure 16a,c,e,g,i,k and Figure 15a,b), although the exoskeleton tracks various human trajectories derived from several human subjects, the control precision can be guaranteed.

Moreover, to verify the online efficiency of the proposed algorithm, the experimental results of the torque tracking performance are given in Figure 16b,d,f,h,j,k and Figure 15c,d. Since the human subjects’ trajectories are different, the driven torque for hip and knee joints is not the same. Consequently, to demonstrate the control performance of rejecting the human disturbance for four human subjects, the RMSE comparison of torque error is presented in Figure 17. The maximum of the RMSE percentage was less than 5.32 for the knee joint, while the maximum of the RMSE percentage was 4.69.

## 5. Conclusions

In applications related to exoskeletons driven with physical HRI, the precision of the physical sensing is still a substantial barrier to enhancing the control performance. In this paper, an elastic module is proposed to improve the compactness of the mechanical structure. Moreover, with its intrinsic sensing property, a corresponding control scheme—so-called evolving internal model control—is introduced. This scheme aims to improve the control precision with compensation from the difference between the exoskeleton and distributed forward model. Additionally, in order to adjust the forward model in real-time, a distributed online evolving model is presented to address this issue and lower the computation expense. From the experimental results, our proposed control scheme can provide more extensive bandwidth and less tracking errors compared with the PD control algorithm. Moreover, the RMSE value of torque errors also indicates a lower output impedance. Thus, the proposed compact mechanical structure and its control algorithm may provide a feasible solution for human–machine interaction applications. Future work will focus on more complicated specific tasks, such as running, left weighting, and whole body assistance.

## Figures and Tables

**Table 1:** Joints ranges of motion.

Joints	DoF	Scope
Hip	Flexion/extension	$-45^{\circ }$–$90^{\circ }$
	Adduction/abduction	$-20^{\circ }$–$35^{\circ }$
	Medial/lateral rotation	$-35^{\circ }$–$35^{\circ }$
Knee	Flexion/extention	$0^{\circ }$–$85^{\circ }$
Ankle	Plantarflexion/dorsiflextion	$-30^{\circ }$–$25^{\circ }$
	Pronation/external rotation	$-25^{\circ }$–$15^{\circ }$
	Inversion/eversion	$-15^{\circ }$–$10^{\circ }$

**Table 2:** Properties of the elastic module.

Parameters	Values
stiffness	60.2 Nm/rad
diameter of outer circle	60 mm
diameter of inner circle	8 mm
maximum torsion torque	4 Nm
thickness	5 mm
maximum deflection	0.087 rad
resolution	0.1 Nm

**Table 3:** NLL comparison of four fusion methods with full Gaussian process (GP).

Methods	Full GP	PoE	gPoE	BCM	rBCM
NLL	−3.12	−3.22	−2.81	−3.25	−2.54

**Table 4:** Comparison of computation cost.

Methods	GP [42]	Sparse GP [51]	Distributed GP [41]	Proposed Method
Training	$O(N^{3})$	$O(NQ^{3})$	$O(MP^{3})$	$O(MP^{3})$ or $O(MP^{2})$
prediction	$O(N^{2})$	$O(NQ^{2})$	$O(MP^{2})$	$O(MP^{2})$

**Table 5:** Human subjects.

Subject	Gender	Age (Years)	Mass (kg)	Height (m)	Status
A	M	25	80	1.83	Healthy
B	M	28	90	1.75	Healthy
C	M	25	70	1.80	Healthy
D	M	26	90	1.80	Healthy

**Table 6:** RSME comparison of three algorithms.

RSME	Control with Disturbance Observer [17]	Force Control for Compact Rotary Series Elastic Actuator [33]	Proposed Control
Hip	Not mentioned	Not mentioned	4.36%
Knee	9%	5.5%	4.73%
